# Single Layer Bismuth Iodide: Computational Exploration of Structural, Electrical, Mechanical and Optical Properties

**DOI:** 10.1038/srep17558

**Published:** 2015-12-02

**Authors:** Fengxian Ma, Mei Zhou, Yalong Jiao, Guoping Gao, Yuantong Gu, Ante Bilic, Zhongfang Chen, Aijun Du

**Affiliations:** 1 School of Chemistry, Physics and Mechanical Engineering, Science and Engineering Faculty, Queensland University of Technology (QUT), Gardens Point Campus, QLD 4001, Brisbane, Australia; 2 CSIRO Manufacturing, Virtual Nanoscience Lab, Parkville 3052 VIC, Australia; 3 Department of Chemistry, Institute for Functional Materials, University of Puerto Rico, San Juan, PR 00931, United States; 4 Department of Physics and State Key Laboratory of Low-Dimensional Quantum Physics, Tsinghua University, Beijing 100084, People’s Republic of China

## Abstract

Layered graphitic materials exhibit new intriguing electronic structure and the search for new types of two-dimensional (2D) monolayer is of importance for the fabrication of next generation miniature electronic and optoelectronic devices. By means of density functional theory (DFT) computations, we investigated in detail the structural, electronic, mechanical and optical properties of the single-layer bismuth iodide (BiI_3_) nanosheet. Monolayer BiI_3_ is dynamically stable as confirmed by the computed phonon spectrum. The cleavage energy (E_*cl*_) and interlayer coupling strength of bulk BiI_3_ are comparable to the experimental values of graphite, which indicates that the exfoliation of BiI_3_ is highly feasible. The obtained stress-strain curve shows that the BiI_3_ nanosheet is a brittle material with a breaking strain of 13%. The BiI_3_ monolayer has an indirect band gap of 1.57 eV with spin orbit coupling (SOC), indicating its potential application for solar cells. Furthermore, the band gap of BiI_3_ monolayer can be modulated by biaxial strain. Most interestingly, interfacing electrically active graphene with monolayer BiI_3_ nanosheet leads to enhanced light absorption compared to that in pure monolayer BiI_3_ nanosheet, highlighting its great potential applications in photonics and photovoltaic solar cells.

Since the discovery of graphene and its excellent electronic/mechanical properties^1^,^2^, tremendous research efforts have been focusing on searching new two-dimensional (2D) materials such as hexagonal boron nitride, transition metal dichalcogenides, and transition metal halides^3^,^4^,^5^,^6^,^7^,^8^,^9^. These 2D materials are bringing revolutions to numerous advanced applications due to their unique and fascinating physical and chemical properties. For example, monolayer MoS_2_ can be used as transistors with room-temperature current on/off ratios of 10^8^. The advantages of 2D materials are so appealing that it is strongly desirable to explore a wide range of 2D materials, other than graphene, to satisfy different purposes. For example, 2D materials with a suitable band gap would meet the needs of field effect transistors or optoelectronic devices^10^,^11^,^12^. More interestingly, a new family of 2D materials, i.e., van der Waals type hetero-structures, can be assembled in a designed manner, which has already proven successful for a number of electronic applications in the area of ultrathin and flexible devices^13^,^14^,^15^,^16^,^17^. Over the past decade, a number of experimental methods have been developed to exfoliate layered materials in order to produce monolayer nanosheets, such as liquid exfoliation that involves oxidation, ion intercalation/exchange, or surface passivation by solvents^18^,^19^.

Some theoretical methods have been developed and employed to search new quasi-two-dimensional (Q2D) materials such as particle swarm optimization (PSO), “atom substitution” and “mechanical exfoliation”. PSO is generally used to locate the global minimum structure. Using PSO, Li *et al.* discovered a novel 2D inorganic material, namely Be_2_C monolayer, in which each carbon atom binds to six Be atoms in an almost planar fashion, forming a quasi-planar hexa-coordinate carbon moiety^20^; Tan *et al.* predicted that the BSi_3_ silicene containing planar cyclic six-membered silicon rings (c-BSi_3_) is the global minimum of BSi_3_ monolayer^21^. Additionally, atom substitution to the common layered structure is used to construct other new types of 2D materials. For instance, by atom substitution of the considered single layer structure, Arunima *et al.* examined the structure, stability, and electronic properties of 2D material in the family of group-IV mono-chalcogenides^22^. “Mechanical exfoliation” is applied to obtain monolayer directly from their layered bulk structures. Zhao *et al.* predicted that the freestanding Ca_2_N monolayer could be exfoliated from the bulk, and therefore obtaining 2D electron gas in free space without resorting to electron doping^13^.

Up to now, a diverse range of intriguing properties in 2D materials have been revealed, highlighting the potential use for important applications in energy^23^, photonics^10^,^24^,^25^ and nanoelectronics^26^,^27^,^28^. However, the practical applications based on 2D materials are still very limited, because they suffer from serious bandgap hurdles, e.g. the lack of obvious gap in graphene^29^ and too large gap in boron nitride^30^. Single-layer transition metal dichalcogenides such as MoS_2_ possess an appropriate bandgap^31^, but are strongly influenced by metal contacts, interface traps, charged impurities, dielectric environment, and structural defects^32^,^33^,^34^. Therefore, the search for new types of 2D structures is of paramount importance for the fabrication of next generation nanodevices.

Bismuth tri-iodide (BiI_3_) is a typical metal halide, the stacking in bulk counterpart is in ABC order with highly ionic Bi-I bond within the layers and weak van der Waals interaction between layers (Fig. 1a). It has been used for room temperature gamma-ray detection, primarily due to its intermediate band gap, high density, and high effective atomic number^35^,^36^. The structural, electronic and optical properties of BiI_3_ crystal have been reported both experimentally and theoretically^37^,^38^,^39^. Podraza *et al.* demonstrated the strong spin-orbital coupling (SOC) effect in bulk BiI_3_^40^. Meanwhile, the BiI_3_ thin films and plates have been synthesized by different approaches such as thermal evaporation^41^, hot wall technique^42^,^43^ and physical vapour deposition^35^. Exploring the BiI_3_ nanostructures is beneficial to gain insights into the properties of BiI_3_ at the atomic scale. So far, single-layer BiI_3_ nanosheet has not been synthesized. Under this context, a systematic theoretical investigation on its structure, stability, electronic, mechanical and optical properties can not only enhance our understanding to their intrinsic characteristics, but also provide useful guidelines for the experimental synthesis of monolayer BiI_3_ and facilitate their practical applications.

In this work, by means of density functional theory (DFT) computations, we first evaluate the stability of monolayer BiI_3_ and the feasibility to exfoliate it from the bulk phase. Subsequently the electronic, mechanical and optical properties of monolayer BiI_3_ nanosheets are investigated. We find that the SOC is significant and can reduce bandgap by around 1.0 eV in monolayer BiI_3_ nanosheet. In addition, the band gap can be modulated with a biaxial strain. Most interestingly, forming a 2D van der Waals type heterostructure by interfacing electrically active graphene with single-layer BiI_3_ nanosheet can significantly enhance the visible light response, i.e., shifting the absorption edge by 2 eV for a hybrid graphene/BiI_3_ nanocomposite compared to that for a pure single BiI_3_ nanosheet, which suggests its potential applications in optoelectronics and photovoltaics.

## Computational details

All the calculations were performed employing the generalized gradient approximation in the Perdew-Burke-Ernzerhof form (GGA-PBE)^44^ and the projector augment wave method^45^,^46^, as implemented in Viena *ab intio* simulation package (VASP)^47^,^48^. A dispersion correction of the total energy (DFT-D3 method)^49^ was used to simulate the long-range van der Waals interaction. The plane-wave energy cutoff was set to 400 eV for geometry optimization and to 500 for static electronic structure and optical property computations. To study 2D systems under the periodic boundary conditions, a vacuum layer with a thickness of at least 20 Å is inserted to avoid the interaction between periodic images. All the geometry structures were fully relaxed until energy and force were converged to 1E^−5^ eV and 0.005 eV/Å, respectively. Unit cell of BiI_3_ (containing 8 atoms) with 5 × 5 × 1, 9 × 9 × 1 and 17 × 17 × 1 Monkhorst−Pack k-point sampling were used for BiI_3_ monolayer geometry optimization, static electronic structure and optical property calculations, respectively. Phonon dispersion analysis was performed using the Phonopy code^50^ interfaced with the density functional perturbation theory^51^ implemented in VASP. In phonon calculations, an increased plane wave energy cutoff of 500 eV and an 11 × 11 × 1 k-point sampling were employed, accompanying with more stringent convergence criteria.

The hybrid graphene/BiI_3_ nanocomposite was simulated using a 1 × 1 unit cell for BiI_3_ which matches well with the 3 × 3 supercell of graphene. The corresponding lattice mismatch is about 2%. In nanocomposite calculations, the k-point mesh used for geometry optimization and static calculation was 5 × 5 and 15 × 15, respectively.

The frequency-dependent dielectric matrix was calculated for the BiI_3_ nanosheet and for the hybrid graphene/BiI_3_ nanocomposite. The imaginary part is determined by a summation over empty states using the equation^52^:


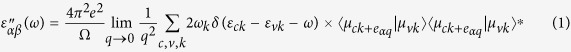


where the indices 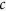 and 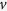 represent conduction and valence band states, respectively. 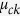 refers to the cell periodic part of the orbitals at the k-point. A large number of empty (conduction band) states are included for the summation in the equation.

## Results and Discussion

### Structure of bulk and monolayer BiI_3_

**Figure 1:**
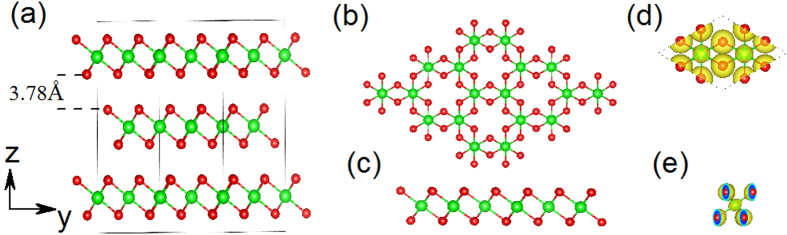
(**a**) Side view of BiI_3_ bulk crystal; (**b**) top and (**c**) side views of BiI_3_ nanosheet; red and green ball represent the iodine and bismuth atoms, respectively; (**d**) top and (**e**) side views of Iso-surface (0.045 ev/au^3^) for electronic density of monolayer BiI_3_.

The stacking order in a bulk Bismuth tri-iodide (BiI_3_) is ABC with highly ionic Bi-I bond within the layers and weak van der Waals interaction between layers as shown in Fig. 1(a). The interlayer distance is 3.38 Å in the bulk BiI_3_. For single layer BiI_3_ (Fig. 1(b,c)), bismuth atom planes are located between top and bottom iodide atomic planes, which forms the sequence I–Bi–I plane. The bond length between bismuth and iodine atom is about 3.1 Å, which is similar to that in the bulk BiI_3_. The charge density distribution, as shown in Fig. 1 (d,e), present the ionic Bi-I bond character in the monolayer BiI_3_.

### Stabilities and feasibility to realize in experiment

**Figure 2:**
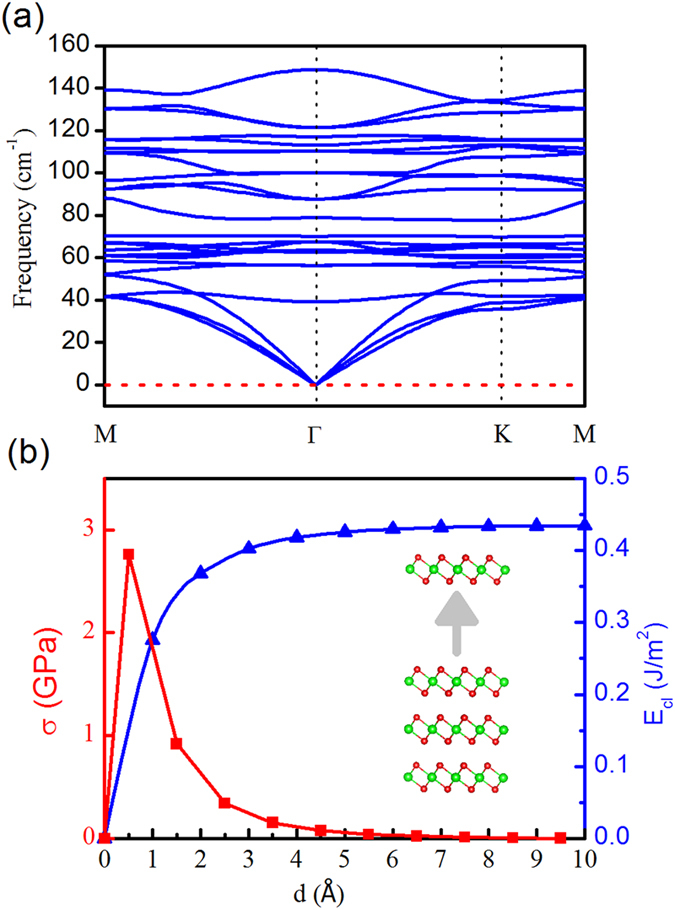
(**a**) Phonon dispersion of BiI_3_ monolayer; (**b**) cleavage energy in J/m^2^ (blue line) and its derivative σ in GPa (red line) as a function of the separation distance *d* for a fracture in BiI_3_ monolayer. Inset: Separating a monolayer from its neighbouring tri-layer.

Before the detailed electronic structure investigations, we firstly examine the dynamic stability of monolayer BiI_3_ nanosheet by calculating its phonon band structure along the high symmetry line from M to Gama to K to M (Fig. 2(a)). Clearly, no imaginary frequency appears in the whole 2D reciprocal space, which confirms the dynamic stability of the BiI_3_ monolayer.

Subsequently, we evaluate the atom binding energy (*E*_*b*_), which is defined as *E*_*b*_=[2*E*(Bi) + 6*E*(I) – *E*(BiI_3_)]/8, where *E*(Bi), *E*(I), and *E*(BiI_3_) are the total energies of bismuth atom, iodine atom, and BiI_3_ sheet, respectively. Based on this definition, systems with stronger binding strength have larger (positive) *E*_*b*_ values. The positive binding energy (2.69 eV per atom) indicates that BiI_3_ monolayer is stable.

We then check the possibility to obtain BiI_3_ monolayer via a mechanical exfoliation strategy. Thermodynamically, the exfoliation process should overcome a cleavage energy *E*_*cl*_, which is determined by the interlayer coupling strength^53^. We simulated the separation of a BiI_3_ monolayer from a neighbouring tri-layer (inset of Fig. 2(b)). The corresponding cleavage energy as a function of distance is shown in Fig. 2(b). The cleavage energy of BiI_3_ (0.43 J/m^2^) is comparable to the experimentally estimated *E*_*cl*_ value of graphite (0.37 J/m^2^). 54 By performing a scan on the separation distance *d* of the fracture, we obtain the theoretical cleavage strength σ, which is defined as the maximum derivative of *E*_*cl*_ (Fig. 2(b))^53^. The calculated cleavage strength is about 2.8 GPa, which is similar to the value of graphite (2.10 GPa). Since graphene^55^ and many other materials^56^ can be exfoliated to obtain 2D atomic crystals, we expect that BiI_3_ is also able to be exfoliated by either Scotch tape or atomic force microscopy tip^57^. Notice that, although the exfoliation process discussed here can be routinely used in laboratory, an effective way to produce BiI_3_ monolayer samples in industry is still an interesting goal to pursue.

### Electronic properties

**Figure 3:**
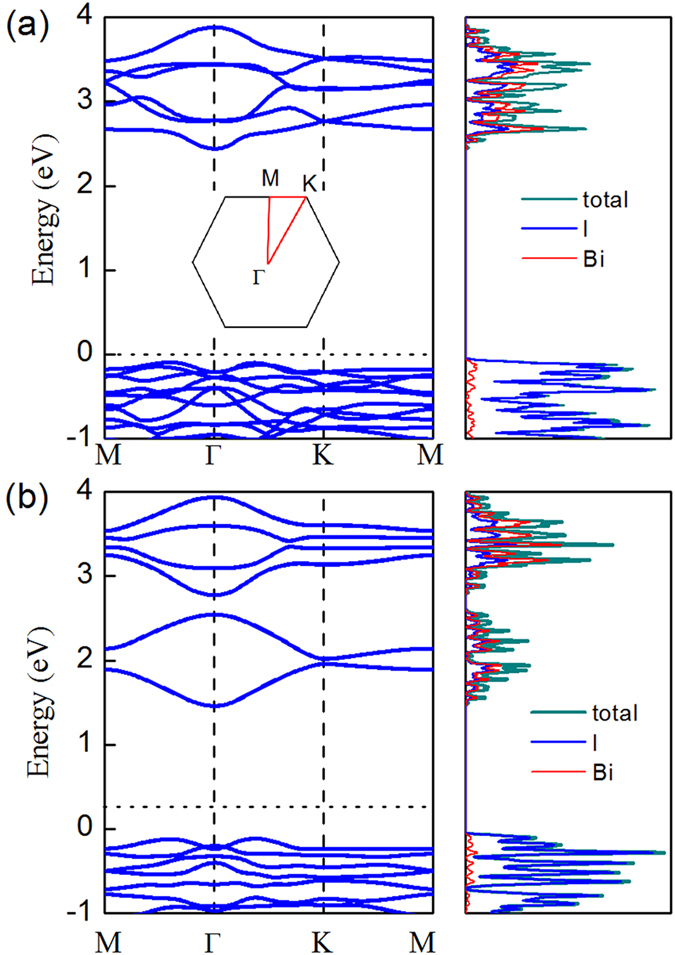
Band structure and total DOS of BiI_3_ monolayer (**a**) without SOC; (**b**) with SOC. Inset is 2D Brillouin zone.

With the optimized monolayer BiI_3_ and confirmed dynamic stability, we now turn to study the ground-state band structure and density of states (DOS) of BiI_3_ monolayer (as shown in Fig. 3).

For comparison, we first check the band structure of bulk BiI_3_. The obtained indirect band gap of bulk BiI_3_ is 1.5 eV with SOC, which is consistent with previous first-principles estimation of 1.55 eV and is also in good agreement with the experimentally-measured band gap of 1.67 eV by Podraza *et al*^40^.

Then, we investigate the electronic properties for the monolayer. Monolayer BiI_3_ nanosheet is found to be an indirect semiconductor with a bandgap of 2.54 eV without SOC, which is slightly larger than that in its bulk counterpart (2.50 eV without SOC). When SOC is included, the obtained indirect band gap for BiI_3_ monolayer is reduced to 1.57 eV. The band gap reduction by the PBE-SOC method is mainly attributed to the downward shift of the conducting band edge due to the SOC effect. As we can see from the DOS (right column in the Fig. 3), the valence band maximum (VBM) is mainly composed of I atom, and the conduction band minimum (CBM) is dominated by the orbitals of Bi atom. Apparently, the SOC effect reduces the energy level in the conducting band for Bi atom.

Therefore, the above results clearly indicate the existence of strong spin orbit effect in monolayer BiI_3_, and the accurate band gap only can be obtained by considering SOC. Note that the band gap of monolayer BiI_3_ (1.57 eV by PBE+SOC method) perfectly matches the ideal band gap value of solar cell materials (1.5 eV). Therefore, we expect that monolayer BiI_3_ may possess an excellent performance in harvesting the visible light.

### Mechanical properties and the control of electronic properties under mechanical strain

Knowledge of the mechanical properties of a material provides important information in the selection of suitable applications. A material must have the required properties to function adequately and must be durable enough for the expected product lifetime. Stress-strain curves are an extremely important graphical measure of a material’s mechanical properties, which provide a preliminary overview of geometrical change during loading testing. In our computations, the strain is added through the change of lattice parameters, the lattice constant *a* of the strained phase is determined by the percentage strain *h* (*a* = *a*_0_ (1 + *h*)), where *a*_0_ is the equilibrium lattice constants at 0% at strain. The *h* values are from 0 to 14% with an interval of 0.8%. To eliminate the artificial effect of the out-of-plane thickness of the simulation box on the stress, we use the second Piola–Kirchhoff stress^58^ to express the 2D forces per length with units of Nm^-1^. The changes of force in various strains indicate the stress–strain relationship.

Figuire 4(a) presents the calculated stress-strain curve of BiI_3_ monolayer under a biaxial tensile strain. Apparently, the relationship between the stress and strain is linear. The ultimate and breaking strength are at the same point, indicating that the BiI_3_ monolayer is a type of brittle material. The ideal strength for the fracture of the BiI_3_ monolayer is around 13%.

It is known that strain has remarkable effects in tailoring electronic, optical and transport properties of 2D semiconductors. Experimentally, the external strain can be applied to 2D materials by various techniques, for example by the mismatch of lattice constant and thermal expansion between the substrate and the film. Kim et al. have shown the possibility of applying nearly 30% strain to graphene by the use of stretchable substrates^59^. Therefore a full analysis of the strain effect on the band gap and electronic structure of single layer BiI_3_ is highly desired.

**Figure 4:**
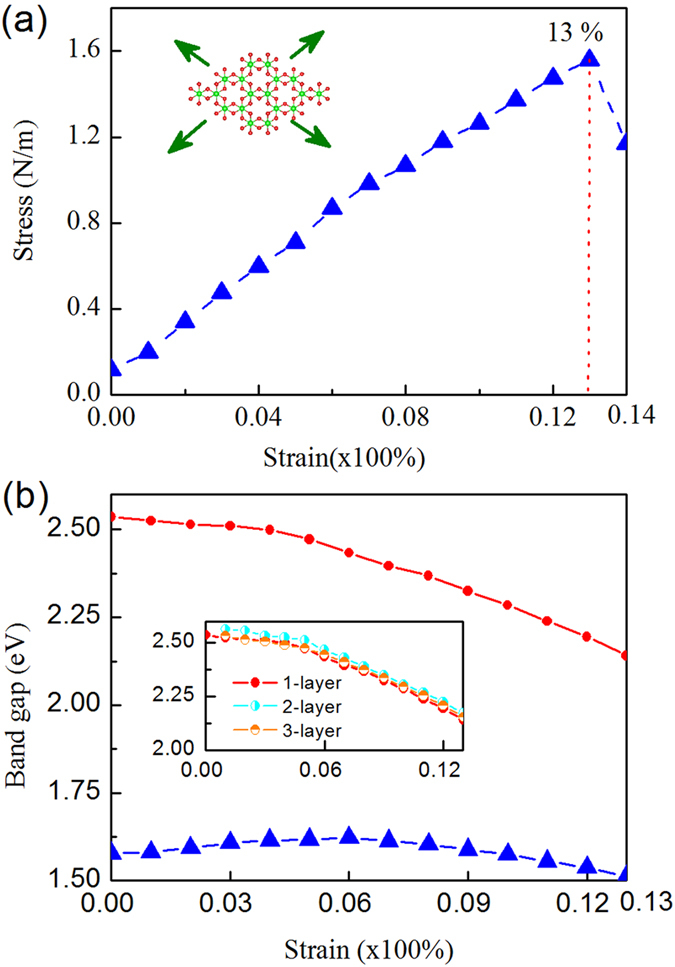
(**a**) Stress-stain curve under biaxial strain. Inset: top view of monolayer BiI_3_ and the directions of strain; (**b**) The band gap at different strain without SOC (red dashed line) and with SOC (blue dashed line); insert: band gap as a function of strain for mono-, bi- and tri- layer BiI_3_.

Figure 4(b) shows the band gap as a function of the biaxial strain, in which the band gap has a downward trend with increasing tensile strain, regardless the SOC is considered or not. Note that the BiI_3_ sheet breaks up at 13%, thus we only study the strain up to this breaking point. With SOC, the change trend of band gap under different strains is moderate, the band gap keeps at 1.57–1.6 eV when the strain is less than 6%, then gradually decreases to 1.5 eV at 13%. Without SOC, the gap slowly decreases from 2.54 eV at 0% to 2.45 eV at 5%, then sharply declines to 2.15 eV at 13%. The detailed band structures of BiI_3_ under different strains are shown in Supporting Information Fig. S1.

Generally, the properties of 2D nanomaterials are dependent on the thickness. Therefore, we evaluated the effect of thickness on the structure and bandgap-strain relationship. In terms of structure parameters and energy (Table 1), the equilibrium lattice constants are gradually reduced from 7.64 Å in monolayer to 7.60 Å in bilayer and then to 7.59 Å in tri-layer. The binding energy per atom (*E*_*b*_) increases from 2.69 eV of monolayer to 2.73 eV of bilayer then to 2.74 eV of tri-layer, which is consistent with favourable interlayer binding energies. The lattice length and binding energy of tri-layer (the thickest layer we studied) are still slightly different with those (7.54 Å and 2.76 eV) of bulk. Considering the demanding computational costs for systems with large thickness, we only evaluated the band gap of strained few-layer BiI_3_ sheets (2 and 3 layers) without SOC to examine the thickness effect. Our DFT computations showed that the band gaps of 2- and 3- layers decrease with the increase of strain (inserted figure in Fig. 4(b)), which is similar with that for monolayer BiI_3._ This indicates the downward trend is irrelevant to the thickness and the interlayer interaction is indeed rather weak. Therefore, BiI_3_ bulk crystals would be an ideal platform to probe 2D electronic property, circumventing the challenge of preparing large-area, single-crystal monolayers.

### Optical properties of BiI_3_ nanosheet and the graphene/BiI_3_ vdW hetero-structure

**Figure 5:**
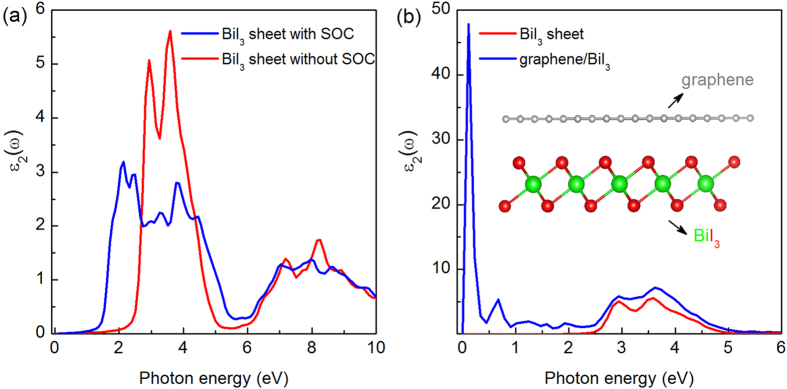
(**a**) The imaginary part of dielectric function ε_2_(ω) of BiI_3_ monolayer without SOC (red line) and with SOC (blue line); (**b**) ε_2_(ω) of BiI_3_ monolayer (red line) and the graphene/BiI_3_ composite (blue line).

The investigation of optical properties can effectively evaluate the performance of a material in light harvesting, which would therefore benefit our exploration of their potential applications in photovoltaics. In the following we calculate the imaginary part of the dielectric function ε_2_(ω) of pure BiI_3_ sheet with SOC and without SOC as shown in the Fig. 5(a). When considering SOC, the first peak of ε_2_(ω) is in between 1.5 and 2.0 eV, which is in agreement with the value (1.57 eV) of the SOC band gap in Fig. 3(b). Similarly, the first peak from the calculation without SOC, located between 2.5 and 3.0 eV, is consistent with the uncorrected band gap shown in Fig. 3(a). The calculated imaginary part of the dielectric functions of BiI_3_ monolayer with SOC shows a red shift about 1.0 eV of absorption edge compared to that without SOC, which indicates the strong effect of SOC to the optical properties of BiI_3_ nanosheet.

Recent experiments have shown that a new family of 2D van der Waals type complex based on the combination of highly conductive graphene and optically active MoS_2_ can generate photon-excite electron-hole pairs within the band gap of MoS_2_, allowing to achieve enhanced photocurrent in visible light region^60^,^61^. To explore this effect, we investigated the geometric structure and optical properties of graphene/BiI_3_ vdW hetero-structure (G/BiI_3_) without SOC effect because it is difficult to include SOC for the large system. For the geometry of the hybrid structure, the graphene covered on BiI_3_ monolayer (insert figure in Fig. 5(b)), has a binding energy of 0.023 eV per atom with an equilibrium interlayer spacing of about 3.67 Å. Therefore, interaction between graphene and the BiI_3_ monolayer is very weak, a typical van der Waals interaction.

As shown in Fig. 5(b), the calculated imaginary part of the dielectric functions of hybrid G/BiI_3_ displays an abrupt peak at 0.1 eV, which demonstrates the red shift of absorption edge is as large as 2.0 eV compared to that for the pristine BiI_3_ nanosheet. The peak at 0.1 eV also indicates the remarkable enhanced abilities to absorb low-energy photons. Furthermore, the hybrid G/BiI_3_ absorbs more photons in visible light region (1.5–3.0 eV) because of the higher ε_2_(ω). Therefore, the hybrid G/BiI_3_ nano-composite is expected to display enhanced photo-catalytic activities under sunlight irradiation.

## Conclusions

In summary, we theoretically investigated the stability, electronic, mechanical and optical properties of BiI_3_ nanosheet by means of DFT computations. BiI_3_ monolayer is dynamically stable, and it is rather feasible to exfoliate the monolayer from the bulk. The BiI_3_ nanosheet is a brittle material with a breaking strain of 13%. The SOC effect is important to compute the band structure of BiI_3_ nanosheet, and the 1.57 eV band gap (with the inclusion of SOC) matches the ideal band gap value of solar cell materials. Moreover, the band gap of BiI_3_ monolayer is strain controllable, and the BiI_3_ films are insensitive to the thickness. Most interestingly, interfacing electrically active graphene with monolayer BiI_3_ nanosheet shows enhanced light absorption compared to that in the pristine monolayer BiI_3_ nanosheet. All these unique and exception properties endow BiI_3_ nanosheet and its composites great potentials for photonics and photovoltaic solar cells.

## Additional Information

**How to cite this article**: Ma, F. *et al.* Single Layer Bismuth Iodide: Computational Exploration of Structural, Electrical, Mechanical and Optical Properties. *Sci. Rep.* **5**, 17558; doi: 10.1038/srep17558 (2015).

## Supplementary Material

Supplementary Information

## Figures and Tables

**Table 1:** The calculated binding energies per atom (*E*_b_, in eV) and equilibrium lattice constants (*a* or *b*, in Å) of BiI_3_ monolayer, 2-layer, 3-layer and bulk.

	Monolayer	Bilayer	Tri-layer	Bulk
*E*_b_	2.69	2.73	2.74	2.76
a or b	7.64	7.60	7.59	7.54
