# Supplementary material for: Single Layer Bismuth Iodide: Computational Exploration of Structural, Electrical, Mechanical and Optical Properties
Source: Sci Rep. 2015 Dec 2;5:17558. doi: 10.1038/srep17558 (PMC4667189; doi:10.1038/srep17558)
Supplement: Supplementary Information [file srep17558-s1.doc]

**Single Layer Bismuth Iodide: Computational Exploration of Structural, Electrical, Mechanical and Optical Properties**

Fengxian Ma,^1^ Mei Zhou,^4^ Yalong Jiao,^1^ Guoping Gao,^1^ Yuantong Gu,^1^ Ante Bilic,^2^ Zhongfang Chen^3^ and Aijun Du^1^, *

*^1^School of Chemistry, Physics and Mechanical Engineering Faculty, Queensland University of Technology, Gardens Point Campus, QLD 4001, Brisbane, Australia*

*^2^CSIRO Manufacturing, Virtual Nanoscience Lab, Parkville 3052 VIC, Australia*

*^3^Department of Chemistry, University of Puerto Rico, Rio Piedras Campus, San Juan, Puerto Rico 00931, United States*

*^4^Department of Physics and State Key Laboratory of Low-Dimensional Quantum Physics, Tsinghua University, Beijing 100084, People’s Republic of China*

*[aijun.du@qut.edu.au](mailto:aijun.du@qut.edu.au)


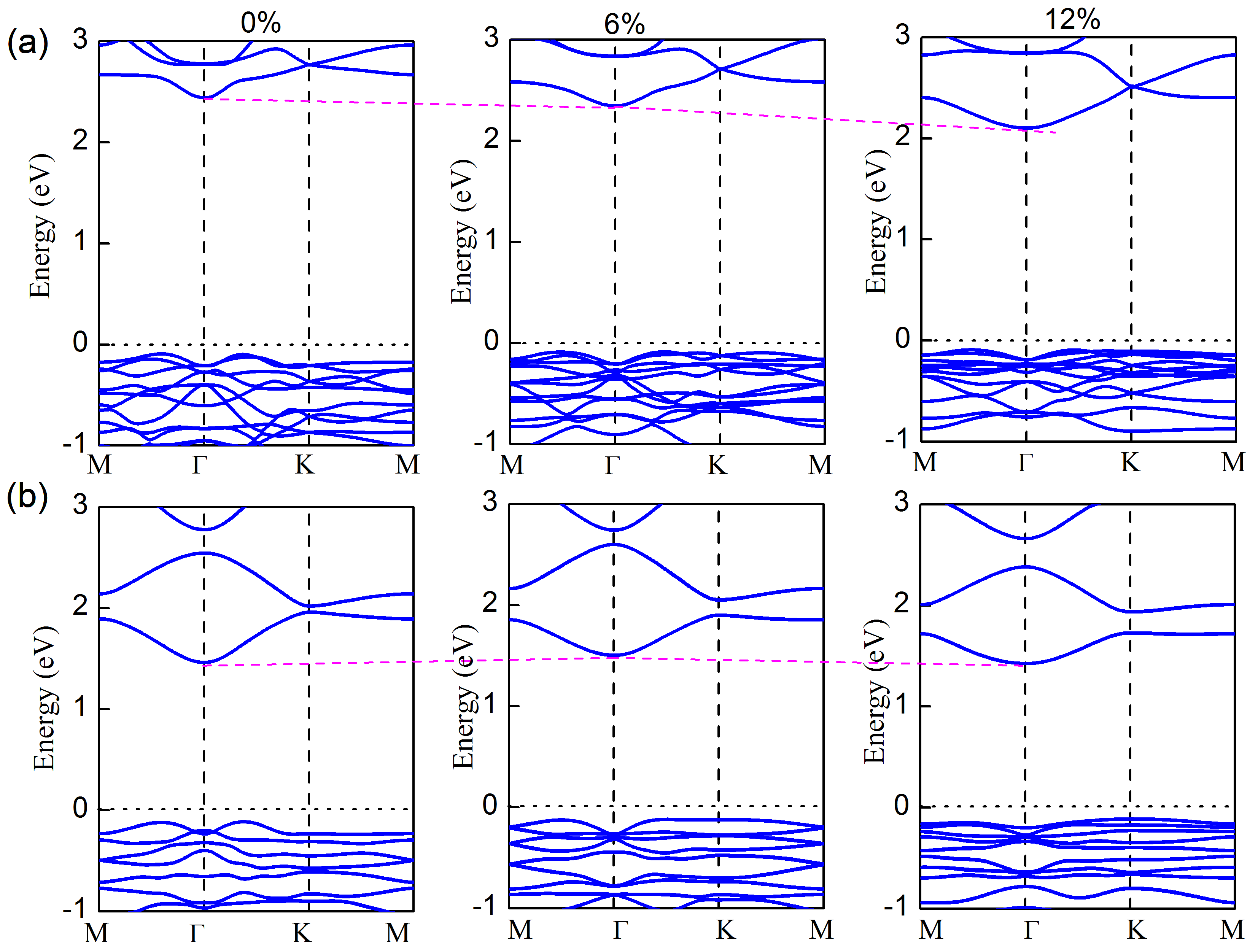


FIG. S1. Band structure of BiI_3_ monolayer under 0%, 6% and 12% strain (a) without SOC; (b) with SOC.
